# The Subtype Identity of Testicular Cancer Cells Determines Their Immunostimulatory Activity in a Coculture Model

**DOI:** 10.3390/cancers15092619

**Published:** 2023-05-05

**Authors:** Fabian A. Gayer, Miriam Henkel, Juliane Luft, Sybille D. Reichardt, Alexander Fichtner, Tobias J. Legler, Holger M. Reichardt

**Affiliations:** 1 Institute for Cellular and Molecular Immunology, University Medical Center Göttingen, 37073 Göttingen, Germany; fabian.gayer@med.uni-goettingen.de (F.A.G.); m.henkel01@stud.uni-goettingen.de (M.H.); juliane.luft@stud.uni-goettingen.de (J.L.); sybille.reichardt@med.uni-goettingen.de (S.D.R.); 2 Clinic of Urology, University Medical Center Göttingen, 37075 Göttingen, Germany; 3 Institute of Pathology, University Medical Center Göttingen, 37075 Göttingen, Germany; alexander.fichtner@med.uni-goettingen.de; 4 Department of Transfusion Medicine, University Medical Center Göttingen, 37075 Göttingen, Germany; tlegler@med.uni-goettingen.de

**Keywords:** testicular germ cell cancer, non-seminoma, seminoma, tumor microenvironment, T cell, monocyte, inflammation, cytokines, TCam-2, NTERA-2

## Abstract

**Simple Summary:**

Testicular germ cell cancer (TGCC) is characterized by an extensive immune cell infiltration, which generates a pro-inflammatory tumor microenvironment (TME). The aim of this study is to compare the interaction of tumor cells representative of two major TGCC subtypes with the immune system in an in vitro coculture model. We found that the non-seminomatous cell line NTERA-2 lacks immunostimulatory capacity and even inhibits T cell and monocyte activity, thus sharply contrasting the opposing properties of the seminomatous cell line TCam-2. We hypothesize that different immunological characteristics of tumor cell subtypes may explain some of the clinical characteristics of TGCC.

**Abstract:**

Testicular germ cell cancer (TGCC) is subdivided into several subtypes. While seminomatous germ cell tumors (SGCT) are characterized by an intensive infiltration of immune cells which constitute a pro-inflammatory tumor micromilieu (TME), immune cells in non-seminomatous germ cell tumors (NSGCT) are differently composed and less abundant. Previously, we have shown that the seminomatous cell line TCam-2 promotes T cell and monocyte activation in a coculture model, resulting in mutual interactions between both cell types. Here we set out to compare this feature of TCam-2 cells with the non-seminomatous cell line NTERA-2. Peripheral blood T cells or monocytes cocultured with NTERA-2 cells failed to secrete relevant amounts of pro-inflammatory cytokines, and significantly downregulated the expression of genes encoding activation markers and effector molecules. In contrast, immune cells cocultured with TCam-2 cells produced IL-2, IL-6 and TNFα, and strongly upregulated the expression of multiple pro-inflammatory genes. Furthermore, the expression of genes involved in proliferation, stemness and subtype specification remained unaltered in NTERA-2 cells during coculture with T cells or monocytes, indicating the absence of mutual interactions. Collectively, our findings uncover fundamental differences between SGCT and NSGCT in their capability to generate a pro-inflammatory TME, which possibly impacts the clinical features and prognosis of both TGCC subtypes.

## 1. Introduction

Influences of the immune system on cancer progression, prognosis and therapy have gained significant attention in recent years. On one hand, chronic inflammation is a major risk factor for tumor development and known to facilitate malignancy [1]. On the other hand, pro- and anti-inflammatory activities of both T and myeloid cells in the tumor microenvironment (TME) influence tumor growth [2,3]. The TME is a complex structure that surrounds the tumor, and not only consists of immune cells, but contains fibroblasts, extracellular matrix, blood vessels, cytokines and hormones [4]. Since mutual interactions between the different cell types and mediators in the TME significantly influence tumor progression, scores based on the intensity and quality of tumor-infiltrating immune cells are increasingly used for risk classification and prognostic evaluation of tumors [5]. Moreover, surface molecules and secreted mediators of immune cells are widely appreciated as important targets of anti-tumor therapies based on monoclonal antibodies, small molecular drugs, checkpoint inhibitors and CAR-T cells [6,7,8]. These innovative concepts mark the onset of a new area of cancer immunotherapy; therefore, more detailed insights into the immunological basis of cancer are urgently needed.

Testicular germ cell cancer (TGCC) is the most frequent tumor of young men, and can be subdivided into seminomatous germ cell tumors (SGCT) and non-seminomatous germ cell tumors (NSGCT) based on their histological and clinical features [9,10]. NSGCT represent a heterogenous group of tumors, including embryonic carcinoma, which is the most undifferentiated subtype, as well as embryonal and post-pubertal teratoma, choriocarcinoma and yolk sack tumors [11]. In general, TGCC stand out amongst cancers due to their excellent prognosis with SGCT, showing an even better overall survival rate and lower risk of recurrent disease courses than NSGCT [12]. These features are associated with the intensive infiltration of SGCT via various immune cells, in particular CD8^+^ cytotoxic T cells, M1 macrophages and a minor fraction of B cells [13,14]. Interestingly, NSGCT are also infiltrated via immune cells, albeit to a lower extent and with a different composition [15]. The TME of SGCT is pro-inflammatory in nature, which is unusual since tumors normally promote an accumulation of regulatory T cells and macrophages with an M2 phenotype [16]. Although there is some evidence that this unusual property of SGCT is related to its good prognosis, the underlying mechanisms remain poorly defined. It is noteworthy that granulomatous inflammation, which is associated with the presence of macrophages and T cells, is often found in SGCT and lymphatic metastasis [17]. The inflammatory foci contain low numbers of vital seminoma cells accompanied by a florid granulomatous reaction, and it is conceivable that there is a connection to reported cases of burned-out seminomas [18]. Besides the infiltrating immune cells, cytokines such as IL-6 are also correlated with tumor progression [6,14,19]. Experiments using an in vitro model revealed that T cells and monocytes sorted from human peripheral blood became activated upon being cocultured with the seminomatous cell line TCam-2, and produced large amounts of IL-6 and TNFα, thus generating a pro-inflammatory TME [20]. Whether this feature is a specific characteristic of the seminomatous phenotype, or shared with other tumor entities, is currently unknown.

TGCC arise from germ cell neoplasia in situ (GCNIS) and further develop to invasive tumors after puberty [21]. Although mechanisms determining tumor development remain vaguely defined, multiple genes have been identified that play a role in this process [22]. Stem cell markers, such as NANOG, OCT3/4 and GDF3, contribute to the maintenance of tumor cell pluripotency, and genes involved in proliferation and cell cycle control, including KI67 and CDK4, promote tumor growth [23]. Animal experiments based on the transplantation of seminomatous cells into mice revealed important influences of the TME on the subtype identity of TGCC cells, depending on whether they were grafted into the testes, flank or brain [24]. These findings confirm that engraftment tumor models are only valid for orthotopic transplantation, and prove that tumor cell differentiation depends on the surrounding TME. Based on these experiments, genes were identified that distinguish between seminomatous and non-seminomatous cells. Most prominently, the pluripotency factor SOX2 is associated with NSGCT, while SOX17 is characteristic of SGCT [25]. The specific features of the different TGCC subtypes are recapitulated using prototypic cell lines entailing mechanistic studies in vitro. NTERA-2 cells are derived from an embryonal carcinoma and show strong similarities with NSGCT, such as high expression of the pluripotency factors SOX2 and OCT3/4 [26,27]. In contrast, TCam-2 cells resemble seminomas, mainly express SOX17 and show solid analogies, for instance, in terms of stem cell marker expression [28,29]. Thus, TCam-2 cells are considered the most reliable seminomatous cell line [28,30]. Using a proteomic approach, several hundred proteins were identified that quantitatively differed between both cell lines [31]. In addition, distinct characteristics were found in their transcriptome and metabolome [32]. These findings indicate that these cell lines are well suited to exploring processes occurring in the TME of each TGCC subtype. Insights into the influences of immune cells on features of TGCC cells have already been obtained via an in vitro coculture model. Here, it was found that the activation of T cells and monocytes resulting from an interaction with TCam-2 cells conversely altered features of the tumor cells [20]. Whether this result is the consequence of immune cell activation and cytokine release, or if T cells and monocytes would induce the same effects without prior activation, has not yet been explored.

Considering the previously observed mutual interactions between TCam-2 cells and sorted immune cells, and the specific clinical features of SGCT and NSGCT, we wondered whether non-seminomatous cells induced immunological changes other than seminomatous cells. Thus, we employed an in vitro model based on coculturing NTERA-2 and TCam-2 cells with either T cells or monocytes sorted from human peripheral blood. It turned out that NTERA-2 cells were able to neither activate T cells and monocytes, nor induce the release of pro-inflammatory cytokines, thus sharply contrasting the properties of TCam-2 cells. Conversely, features of NTERA-2 cells, such as growth and stemness, remained unaltered in coculture. Hence, TGCC subtypes differ in their immunomodulatory capacity, which can also be expected to impact their clinical features.

## 2. Materials and Methods

### 2.1. Culturing of Tumor Cell Lines

The cell lines employed in this work were maintained in DMEM/Ham’s F-12 medium with L-Glutamine (Capricorn Scientific, Ebsdorfergrund, Germany), 10% FCS and 1% penicillin/streptomycin at 37 °C and 5% CO_2_, and split twice a week at a ratio of 1:3. The human non-seminomatous cell line NTERA-2 was originally obtained from ATCC and is reminiscent of an embryonal carcinoma [33]. The human seminoma-derived cell line TCam-2 was provided by Daniel Nettersheim (University of Düsseldorf, Germany) and previously used in our laboratory [20]. The initial passage number of both cell lines was 4, and another 5–10 passages were performed in the course of the experiments.

### 2.2. Isolation of T Cells and Monocytes from Human Buffy Coats

Buffy coats were collected from healthy volunteers by the Department of Transfusion Medicine of the University Medical Center Göttingen, Germany in the course of routine blood donations. PBMC were purified via density gradient centrifugation using Lymphoprep^TM^ (Stemcell Technologies, Cologne, Germany), as described previously [20]. T cells or monocytes were then sorted with the help of the EasySep^TM^ human T cell Isolation Kit or EasySep^TM^ Monocyte Enrichment/ Isolation Kit (Stemcell Technologies), according to the instructions of the manufacturer. The purity of the cell preparations was tested via flow cytometry, employing anti-hCD3 (T cells) or anti-hHLA-DR (monocytes) antibodies, and found to be always >95%.

### 2.3. Coculture Experiments

NTERA-2 and TCam-2 cells were cultured in a volume of 2 mL of medium for 24 h in 6-well plates starting with 1.5 × 10^5^ cells, as previously described [20]. The next day, T cells (3 × 10^6^ cells/well) or monocytes (2 × 10^6^ cells/well) were added to the tumor cells and cocultured in a total volume of 4 mL for another 24 h at 37 °C and 5% CO_2_. Immune and tumor cells were also cultured individually as controls. T cells and monocytes were collected through carefully aspirating them together with the medium from the monolayers of NTERA-2 or TCam-2 cells. Subsequently, the harvested immune cells were centrifuged for 7 min at 350× *g*, and the cell pellets and the supernatants were stored separately. Routine analysis via flow cytometry revealed a purity of 95–99% for T cells and 86–94% for monocytes. Adherent NTERA-2 or TCam-2 cells were dispersed via incubation with 0.1% trypsin-EDTA (ThermoFisher, Waltham, MA, USA) for 5 min. The tumor cells were then washed and stored for analysis.

### 2.4. Flow Cytometry

T cells and monocytes were stained with fluorochrome-conjugated monoclonal antibodies obtained from BioLegend (Uithoorn, The Netherlands) and analyzed with a FACS Canto II device (BD Bioscience, Heidelberg, Germany). Antibodies recognizing the following antigens were used in this study (clone names in brackets): hCD3 (HIT3a), hCD4 (OKT4), hCD8α (HIT8a), hHLA-DR (I.243), hCD14 (HCD14), hCD25 (BC96) and hCD163 (GHI/61). FlowJo^®^ software (Tree Star, Ashland, OR, USA; version 10.7) was employed for data processing.

### 2.5. ELISA

Cytokine levels in cell culture supernatants were measured with the help of commercially available ELISA kits detecting IL-2, IL-6 or TNFα, following the instructions of the manufacturer (Biolegend). Samples were pre-diluted, depending on the experimental setup, to account for the different cytokine concentrations in cocultures of T cells and monocytes with the two tumor cell lines. Absorbances at 450 nm and 570 nm were recorded using a BioTek Power wave 340 Plate Reader (BioTek Instruments, Germany, Wetzlar).

### 2.6. Quantitative RT-PCR

Total RNA was isolated with the Quick-RNA MiniPrep kit (Zymo Research, Irvine, CA, USA) and used to synthesize cDNA with the iScript kit (Bio-Rad, Munich, Germany). Quantitative RT-PCR (RT-qPCR) analysis was performed on an ABI 7500 Instrument with the SYBR Green Master Mix (both from ThermoFisher). Gene expression was calculated using the ΔΔCt method and normalized to the housekeeping gene *18SRNA*. All primers were synthesized via Metabion (Planegg, Germany); their sequences can be found in Table S1.

### 2.7. Statistical Analysis

All data were depicted as scatter dot plots with bars representing the mean, and were analyzed either via unpaired *t*-test (two groups) or one-way ANOVA, followed by Newman–Keuls Multiple Comparisons test (three groups). GraphPad Prism^®^ software (San Diego, CA, USA; version 9.4) was used for data analysis. Levels of significance are as follows: *: *p* < 0.05; **: *p* < 0.01; ***: *p* < 0.001; n.s.: *p* > 0.05.

## 3. Results

### 3.1. NTERA-2 and TCam-2 Cells Differ in Their Capability to Activate T Cells and Monocytes in Coculture

**Figure 1:**
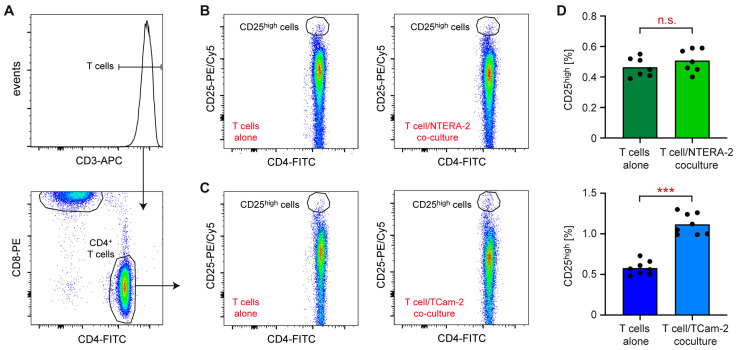
Flow cytometric analysis of T cells following coculture with NTERA-2 or TCam-2 cells. T cells were isolated from buffy coats of healthy volunteers and either cultured alone or cocultured with NTERA-2 or TCam-2 cell for 24 h. Thereafter, T cells were collected and stained with fluorochrome-conjugated antibodies. (**A**) Exemplary analysis of T cells from an NTERA-2 coculture based on CD3, CD4 and CD8 expression. (**B**,**C**) Identification of CD25^high^ cells amongst T cells cultured alone and T cells cocultured with NTERA-2 or TCam-2 cells. Exemplary dot plots are shown for one sample of each experimental group. (**D**) Quantification of CD25^high^ cells in cocultures of T cell with either NTERA-2 or TCam-2 cells compared with monocultures of T cells. N = 7 (NTERA-2), N = 8 (TCam-2). PBMC used in this experiment were collected from a total of five blood donors. Data are depicted as bars representing mean and dots for each individual sample. An unpaired t-test was used for statistical analysis (***: *p* < 0.001, n.s., non-significant with *p* > 0.05).

We previously showed that TCam-2 cells induce an activated phenotype in cocultured immune cells [20]. Here, we asked whether this feature was shared with a cell line representative of another TGCC subtype. To address this question, the non-seminomatous cell line NTERA-2 and the seminomatous cell line TCam-2 cells were cultured for 24 h. The next day, T cells were sorted from the peripheral blood of healthy donors, and either cultured alone for 24 h or cocultured separately with each tumor cell line. Flow cytometric analysis of CD3^+^ CD4^+^ T cells expressing high CD25 surface levels indicative of T cell activation revealed no changes in cocultures with NTERA-2 cells, while their percentage was significantly increased in cocultures with TCam-2 cells (Figure 1A–D).

**Figure 2:**
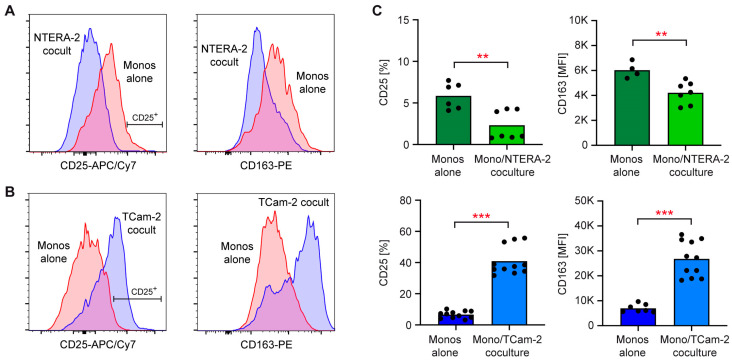
Flow cytometric analysis of monocytes cocultured either with NTERA-2 or TCam-2 cells. Monocytes were sorted from blood donations of healthy volunteers, and then either cultured alone or added to monolayers of NTERA-2 or TCam-2 cells and cocultured for 24 h. Afterwards, monocytes were harvested and stained with fluorochrome-conjugated antibodies. (**A**,**B**) Representative histograms of CD25 and CD163 stainings of CD14^+^ HLA-DR^+^ monocytes cultured alone and cocultured with NTERA-2 or TCam-2 cells. (**C**) Comparison of percentage of CD25^+^ cells and mean fluorescence intensity (MFI, K = 1000-fold) of CD163 between monocytes cultured alone and monocytes cocultured with NTERA-2 or TCam-2 cells. N = 7 (NTERA-2), N = 7 (TCam-2). PBMC were obtained from five blood donors in total. Data are depicted as bars representing mean and dots for each individual sample. Statistical analysis was performed using an unpaired *t*-test (**: *p* < 0.01; ***: *p* < 0.001).

Next, we sorted monocytes from buffy coats of healthy donors and cocultured them with each cell line. Analysis of HLA-DR^+^ CD14^+^ monocytes via flow cytometry revealed a significant downregulation of the activation markers CD25 and CD163 on monocytes collected from cocultures with NTERA-2 cells (Figure 2A,C). In contrast, the levels of both proteins were increased on the surface of monocytes that were cocultured with TCam-2 cells (Figure 2B,C). Collectively, these observations reveal opposite responses of immune cells to NTERA-2 compared to TCam-2 cells. While the seminomatous cell line TCam-2 induces immune cell activation, the non-seminomatous cell line NTERA-2 has no effect on T cells, and even represses monocyte activation.

### 3.2. T Cells and Monocytes Fail to Secrete Pro-Inflammatory Cytokines in Cocultures with NTERA-2 Cells

**Figure 3:**
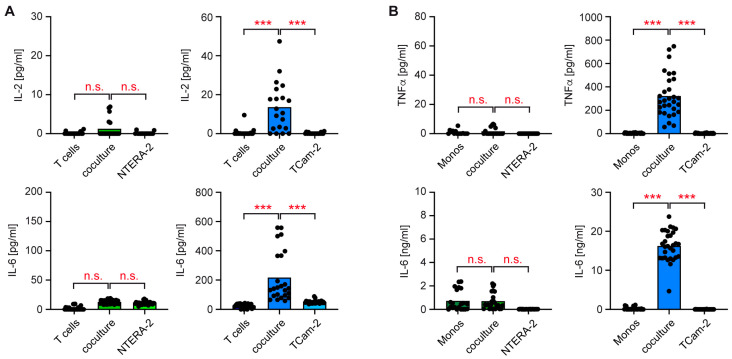
Quantification of cytokine secretion by T cells and monocytes cocultured either with NTERA-2 or TCam-2 cells using ELISA. T cells or monocytes were sorted from blood donations of healthy volunteers, added to monolayers of NTERA-2 or TCam-2 cells and cocultured for 24 h. Individual cell types cultured alone served as controls. Concentration of each cytokine in cell culture supernatant was determined via ELISA. (**A**) IL-2 and IL-6 levels in supernatants of T cell cocultures with NTERA-2 or TCam-2 cells, or in cultures of each cell type cultured alone. N = 16–28 (NTERA-2), N = 19–24 (TCam-2). PBMC were obtained from 6–14 blood donors. (**B**) TNFα and IL-6 levels in supernatants of monocytes cocultured with NTERA-2 or TCam-2 cells, or in cultures of each cell type cultured alone. N = 21–26 (NTERA-2), N = 30–32 (TCam-2). PBMC were obtained from 7–13 blood donors. Data are depicted as bars representing mean and dots for each individual sample. One-way ANOVA, followed by a Newman–Keuls Multiple Comparisons test was used for statistical analysis (***: *p* < 0.001; n.s.: *p* > 0.05).

One of the most prominent consequences of immune cell activation is enhanced cytokine secretion [34]. Hence, we tested whether the different activation states of T cells and monocytes in cocultures with NTERA-2 and TCam-2 cells were also reflected by their cytokine production. T cells and monocytes isolated from the peripheral blood of healthy volunteers were individually cocultured with NTERA-2 or TCam-2 cells, as in the previous experiment. Tumor and immune cells cultured alone served as controls. After 24 h, the cell culture supernatants were harvested, and the levels of pro-inflammatory cytokines were quantified via ELISA. IL-2 and IL-6 were essentially undetectable in cocultures of T cells with NTERA-2 cells, which resembled the monocultures of each cell type (Figure 3A). In contrast, secretion of both cytokines was significantly increased using T cells cocultured with TCam-2 cells compared to both cell types cultured alone (Figure 3A).

Subsequently, we performed a similar analysis for monocytes cocultured with both tumor cell lines. NTERA-2 cells also failed to stimulate cytokine secretion by cocultured monocytes, while TCam-2 cells induced the release of large quantities of TNFα and IL-6 (Figure 3B). It is noteworthy that the amount of IL-6 secreted by monocytes cocultured with TCam-2 cells was around 70-fold higher than by T cells cocultured with TCam-2 cells. Collectively, our data provide evidence that non-seminomatous cells lack the capability to promote a pro-inflammatory microenvironment when exposed to immune cells

### 3.3. The Individual Response of T Cells and Monocytes to TGCC Subtypes Is Linked to Their Differential Gene Expression

**Figure 4:**
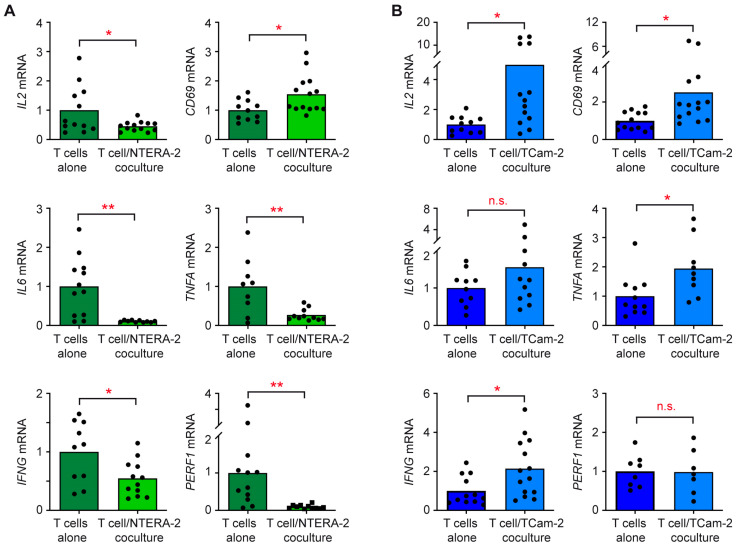
Gene expression analysis of T cells in cocultures with NTERA-2 or TCam-2 cells. T cells were sorted from blood donations of healthy volunteers, added to monolayers of (**A**) NTERA-2 or (**B**) TCam-2 cells and cocultured for 24 h. T cells cultured alone served as controls. Subsequently, total RNA was prepared from T cells harvested from both cultures, reverse transcribed into cDNA and analyzed via RT-qPCR. Relative mRNA levels of *IL2*, *CD69*, *IL6*, *TNFA*, *IFNG* and *PERF1* are depicted as bars representing mean and dots for each individual sample. Housekeeping gene *18SRNA* was used for normalization, and mRNA levels in monocultured T cells were arbitrarily set to 1. N = 10–14 (NTERA-2), N = 8–14 (TCam-2). PBMC employed in this study were obtained from seven blood donors. Data were analyzed via unpaired *t*-test (*: *p* < 0.05; **: *p* < 0.01; n.s., non-significant with *p* > 0.05).

The differences between NTERA-2 and TCam-2 cells concerning surface levels of activation markers and cytokine concentrations in cell culture supernatants could be caused through differences in gene expression, or linked to post-transcriptional mechanisms. To address this issue, we initially analyzed mRNA levels in T cells either cultured alone or together with the tumor cell lines. Expression of the four cytokine genes *IL2*, *IL6*, *TNFA* and *IFNG* in T cells cocultured with NTERA-2 cells was significantly lower than in monocultured T cells (Figure 4A). A similar observation was made for *PERF1* encoding the cytotoxic T cell effector molecule perforin 1. The only exception was the activation marker *CD69*, which was slightly upregulated in cocultured T cells (Figure 4A). In sharp contrast, T cells cocultured with TCam-2 cells expressed significantly increased mRNA levels of *IL2*, *IL6*, *TNFA*, *IFNG* and *CD69* compared to T cells cultured alone; only *PERF1* expression was unaltered (Figure 4B). These findings indicate that NTERA-2 cells oppose a pro-inflammatory gene expression profile in T cells.

**Figure 5:**
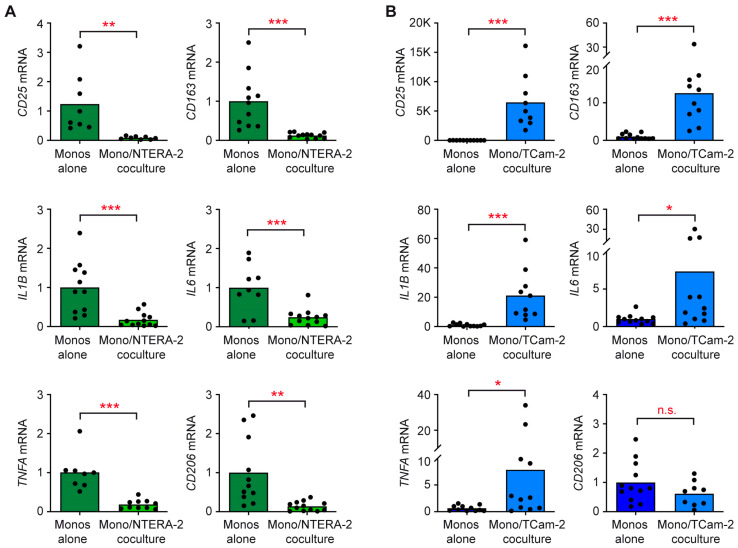
Analysis of gene expression in monocytes cocultured with NTERA-2 or TCam-2 cells. Monocytes were sorted from blood donations of healthy volunteers, added to monolayers of (**A**) NTERA-2 or (**B**) TCam-2 cells and cocultured for 24 h. Monocytes cultured alone served as controls. Subsequently, total RNA was prepared from monocytes collected from both cultures, reverse transcribed into cDNA and analyzed via RT-qPCR. Gene expression of *CD25*, *CD163*, *IL1B*, *IL6*, *TNFA* and *CD206* is represented as bars corresponding to mean and dots for each individual sample. Housekeeping gene *18SRNA* was used to normalize mRNA levels, which were arbitrarily set to 1 in monocytes cultured alone. N = 8–12 (NTERA-2), N = 11–12 (TCam-2). The PBMC were purified from buffy coats of seven donors. Data analysis was performed via unpaired *t*-test (*: *p* < 0.05; **: *p* < 0.01; ***: *p* < 0.001; n.s., non-significant with *p* > 0.05).

Subsequently, we investigated whether the influence of NTERA-2 cells and TCam-2 cells on monocytes differed. To this end, monocytes were either cocultured with the two tumor cell lines for 24 h or cultured alone, followed by RT-qPCR analysis. Expression levels of the activation marker genes *CD25* and *CD163*, the pro-inflammatory cytokine genes *IL1B*, *IL6* and *TNFA*, and the gene encoding the scavenger receptor *CD206*, were significantly downregulated in monocytes cocultured with NTERA-2 cells compared to monocytes cultured alone (Figure 5A). In contrast, all genes except for *CD206* became strongly induced when monocytes were cocultured with TCam-2 cells (Figure 5B). Consequently, NTERA-2 cells prevent monocytes from acquiring a pro-inflammatory gene expression profile. Taken together, our data suggest that TCam-2, but not NTERA-2, cells induce T cell and monocyte activation in coculture through controlling their gene expression profile, resulting in a pro-inflammatory TME in the case of the seminomatous, but not non-seminomatous, cell line.

### 3.4. T Cells and Monocytes Do Not Exert Any Influence on NTERA-2 Cells in Coculture

**Figure 6:**
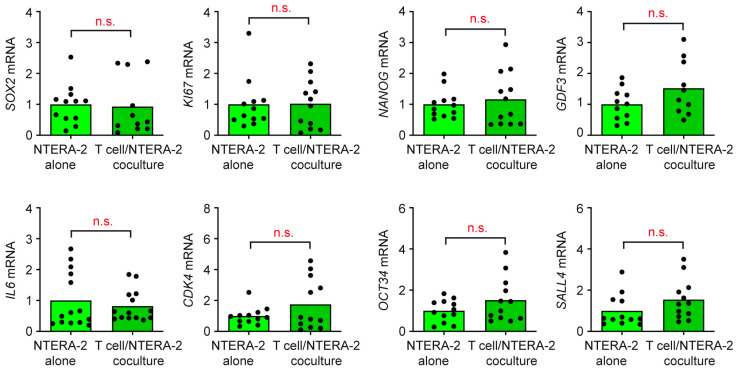
Gene expression analysis of NTERA-2 cells in T cell cocultures. T cells were purified from buffy coats of healthy blood donors and subsequently cocultured for 24 h with NTERA-2 cells. Monocultures of NTERA-2 cells were performed as controls. Total RNA was prepared from NTERA-2 cells collected from either cocultures or monocultures, reverse transcribed into cDNA and analyzed via RT-qPCR. Relative mRNA levels of *SOX2*, *IL6*, *KI67*, *CDK4*, *NANOG*, *OCT34*, *GDF3* and *SALL4* are depicted as bars representing mean and dots for each individual sample. Gene expression was calculated via normalization to housekeeping gene *18SRNA*, and mRNA levels in NTERA-2 cell monocultures were arbitrarily set to 1. N = 12. PBMC from 6 buffy coats were included in this experiment. Data analysis was achieved through employing an unpaired *t*-test (n.s., non-significant with *p* > 0.05).

**Figure 7:**
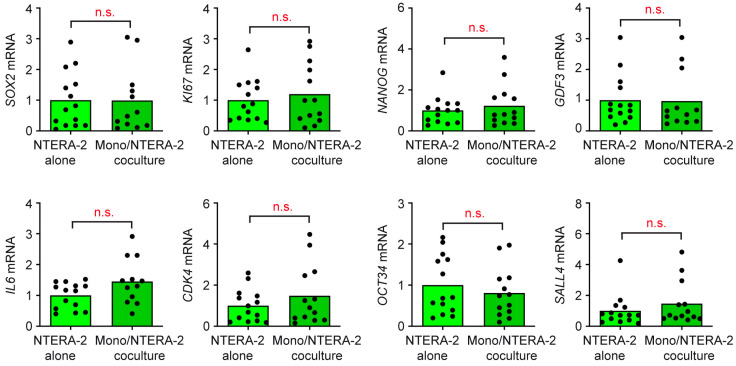
Quantification of gene expression in NTERA-2 cells cocultured with monocytes. Monocytes were sorted from buffy coats of healthy volunteers and cocultured with NTERA-2 cells for 24 h. As a control, NTERA-2 cells were cultured alone. Total RNA was prepared from NTERA-2 cells either collected from cocultures or monocultures, reverse transcribed into cDNA, and analyzed via RT-qPCR. Relative mRNA levels of *SOX2*, *IL6*, *KI67*, *CDK4*, *NANOG*, *OCT34*, *GDF3* and *SALL4* are depicted as bars representing the mean and dots for each individual sample. Housekeeping gene *18SRNA* was used for normalization of mRNA levels, and gene expression in NTERA-2 cell monocultures was arbitrarily set to 1. N = 13–14. PBMC employed in this experiment were collected from seven blood donors. Statistical analysis was performed via unpaired *t*-test (n.s., non-significant with *p* > 0.05).

We had previously found that the activated phenotype of T cells and monocytes, as well as enhanced cytokine secretion, resulted in an altered gene expression profile of cocultured TCam-2 cells. Therefore, we asked whether immune cells influence NTERA-2 cells, even though they do not become activated by them. To this end, NTERA-2 cells were cocultured for 24 h with either T cells or monocytes purified from buffy coats of healthy donors, or cultured alone. Subsequently, a gene expression analysis via RT-qPCR was performed for the non-seminoma marker *SOX*2; the cytokine *IL6*, which is moderately produced also through tumor cells; and the proliferation and cell cycle genes *KI67* and *CDK4*. Furthermore, we tested the stem cell markers *NANOG*, *OCT3/4*, and *GDF3*, as well as *SALL4*, which is a novel diagnostic TGCC marker. Importantly, the expression of all eight genes remained unaltered in NTERA-2 cells, regardless of whether they were cultured alone or cocultured with T cells (Figure 6) or monocytes (Figure 7). This observation confirms that immune cell activation is a prerequisite for their capability to conversely impact features of TGCC cells.

## 4. Discussion

TGCC can be subdivided into SGCT and NSGCT, with the latter being a heterogenous group that includes embryonic carcinoma [10]. An unusual feature of SGCT is their extensive pro-inflammatory TME [13,14,15]. Interestingly, most tumors found in other organs tend to promote the infiltration of regulatory T cells and myeloid-derived suppressor cells, and, additionally, induce the polarization of macrophages towards an anti-inflammatory M2 phenotype [3]. In sharp contrast, SGCT are densely infiltrated with cytotoxic CD8^+^ T cells, M1 macrophages and B lymphocytes [14,15]. Moreover, they express effector molecules, which are destined to kill tumor cells, including perforin 1, granzyme B and TNFα, as well as multiple pro-inflammatory cytokines that are characteristic for activated macrophages and Th1 cells, including IL-1β, IL-6, IL-2 and IFNγ [14]. Th2 cytokines, however, are largely unaltered in SGCT compared to normal testes. It is noteworthy that the multifarious cytokine IL-6, which has been attributed tumor-promoting as well as anti-tumorigenic properties [35], was found to be strongly upregulated in TGCC tissue samples and cell culture models [14,36]. Although TGCC are always infiltrated to a certain degree by immune cells, the TME is differently composed depending on the tumor subtype [15]. The percentage of CD3^+^ T cells in NSGCT is lower than in SGCT, and the relative abundance of regulatory T cells is increased, thus creating a TME in NSGCT that has even partial anti-inflammatory properties [15]. Importantly, this feature is also reflected in the gene expression profiles of both tumor subtypes [15]. NSGCT express lower levels of the T cell-specific gene CD3 and the T cell chemoattractant CCL19. Furthermore, the effector molecules granzyme A, perforin 1 and IFNγ, as well as the transcription factor EOMES, which is predominantly found in CD8^+^ cytotoxic T cells, are all expressed at lower levels in NSGCT compared to SGCT. Finally, the chemokine CXCL9, which is produced by inflammatory macrophages to recruit T cells, and cytokine IL-12, which polarizes T cells towards the Th1 phenotype, are downregulated in NSGCT compared to SGCT. These data suggest that a pro-inflammatory TME is a specific feature of SGCT and, thereby, markedly differs from the immunological features of NSGCT.

It is currently unknown why the immunological properties of SGCT and NSGCT are so different. We now speculated that tumor cells of each subtype differently interact with immune cells. To challenge this hypothesis, we cocultured NTERA-2 and TCam-2 cells with sorted immune cells from the peripheral blood of healthy volunteers. This approach allowed us to evaluate whether both cell lines differed in their capacity to activate T cells and monocytes and generate a pro-inflammatory TME. The results of our flow cytometric analysis showed that NTERA-2 cells failed to induce T cell activation in coculture, and even dampened monocyte activity. Accordingly, the levels of pro-inflammatory cytokines, such as IL-2, TNFα and IL-6, were unaltered in T cell and monocyte cocultures with NTERA-2 cells. In contrast, they were increased in cocultures with TCam-2 cells, which is in line with a previous report from our group [20]. We conclude that seminomatous cells generate a pro-inflammatory TME whereas non-seminomatous cells propagate an anti-inflammatory state. It is noteworthy that IL-6 levels were extremely high in cocultures of TCam-2 cells with monocytes, indicating that this cytokine may play a prominent role in shaping the TME of SGCT, as suggested previously [14,35]. Our gene expression analysis uncovered further differences between SGCT and NSGCT concerning their capability to influence immune cells. Regardless of whether T cells or monocytes were cocultured with NTERA-2 cells, activation markers, pro-inflammatory cytokines and effector molecules were downregulated, presumably leading to an inhibition of the immune cells. In contrast, TCam-2 cells caused an upregulation of the respective genes, supporting our previous conclusion that seminomatous cells promote a pro-inflammatory TME [20]. Furthermore, genes linked to the tumorigenic potential of NTERA-2 cells remained unaltered in both immune cell cocultures. Thus, the two cell lines fundamentally differ in their mutual interactions with T cells and monocytes, which represent crucial components of the TME of every tumor entity.

We have chosen TCam-2 and NTERA-2 cells to elaborate immunological differences between SGCT and NSGCT, rather than other TGCC cell lines, because, in our view, they best reflect the molecular and clinical features of the respective tumor subtypes. SEM-1 cells, for instance, represent an intermediate between both tumor entities, and are derived from an extragonadal tumor, while the cell line JKT-1 lacks the typical over-representation of chromosome 12p found in most SGCT [37,38]. Similarly, the NCCIT cell line does not ideally reflect the characteristics of NSGCT, as it carries a p53 mutation and originates from the mediastinum [33]. We are aware that using only one exemplary cell line for each TGCC subtype represents a certain limitation of our work, and that the conclusions are formally restricted to the comparison between NTERA-2 and TCam-2 cells. It will, thus, be necessary to confirm our in vitro data in the future by studying TGCC patients. In fact, preliminary results of a gene expression analysis of fresh tumor tissue provided evidence that the mRNA levels of *IL2*, *IFNG*, *IL6* and *TNFA* are increased in SGCT but not in NSGCT, which is in line with our cell culture data (unpublished results). We are, therefore, confident that the conclusions drawn from the analysis of NTERA-2 and TCam-2 cells will also hold true in the clinical setting.

In rare cases of TGCC, the primary tumor spontaneously regresses and becomes replaced by fibrotic tissue [39]. These lesions, which are commonly found in metastatic patients, are referred to as burned out testicular tumors (BOTT). Individual studies indicate that BOTT are more often associated with SGCT than NSGCT metastasis [39]. The mechanism underlying this phenomenon, however, is poorly understood. It is conceivable that the pro-inflammatory TME contributes to the eradication of the primary tumor in BOTT patients, and concomitantly promotes the metastatic potential of the tumor cells, thus resulting in an extragonadal manifestation of TGCC. The observation that BOTT are more frequently found in SGCT than NSGCT would be in accordance with the predominant pro-inflammatory TME in this tumor subtype, as characterized by a strong infiltration of CD8^+^ cytotoxic T cells and high levels of pro-inflammatory cytokines, possibly contributing to the regression of the primary testicular tumor.

Overall, our results revealed highly relevant differences concerning the interaction between TGCC subtypes and immune cells, leading to divergent immunological responses and changes in the tumor cell phenotype. With this knowledge in mind, the characteristics of the TME and immunological signature of TGCC should be considered as diagnostic criteria in the future, and potentially used as risk stratification factors, for instance, in terms of relapse rates and the metastatic potential of the tumor. Regarding potential immunomodulatory treatment options, our results entail efforts to promote immune cell infiltration and supply recombinant cytokines, such as IL-6 or TNFα in NSGCT, thereby potentially improving the prognosis of the respective patients. It appears to us that generating a pro-inflammatory TME is instrumental for therapeutic success.

## 5. Conclusions

Subgroups of TGCC differ in their clinical properties and prognosis. The results of our in vitro coculture model suggest that these features could be linked to the immunological characteristics of the TME in each subtype. While the non-seminomatous cell line NTERA-2 suppressed markers of T cell and monocyte activation, and failed to induce the secretion of pro-inflammatory cytokines, the seminomatous cell line TCam-2 strongly activated immune cells and caused the release of large quantities of several pro-inflammatory cytokines. These data suggest that NSGCT in patients may also lack the ability to activate infiltrating immune cells, thereby resulting in the failure to produce pro-inflammatory cytokines and alter crucial properties of the tumor cells. This difference may eventually explain the poorer prognosis of NSGCT and their higher metastatic potential.

## Data Availability

All data are available upon reasonable request.

## Supplementary Materials

The following supporting information can be downloaded at: https://www.mdpi.com/article/10.3390/cancers15092619/s1, Table S1: Primer sequences.

## References

[1] Shacter E., Weitzman S.A. (2002). Chronic inflammation and cancer. Oncology.

[2] Pearce H., Hutton P., Chaudhri S., Porfiri E., Patel P., Viney R., Moss P. (2017). Spontaneous CD4(+) and CD8(+) T-cell responses directed against cancer testis antigens are present in the peripheral blood of testicular cancer patients. Eur. J. Immunol..

[3] Katsuta E., Rashid O.M., Takabe K. (2020). Clinical relevance of tumor microenvironment: Immune cells, vessels, and mouse models. Hum. Cell.

[4] Diez-Torre A., Silvan U., Diaz-Nunez M., Arechaga J. (2010). The role of microenvironment in testicular germ cell tumors. Cancer Biol. Ther..

[5] Ladanyi A. (2015). Prognostic and predictive significance of immune cells infiltrating cutaneous melanoma. Pigment. Cell Melanoma Res..

[6] Kampan N.C., Xiang S.D., McNally O.M., Stephens A.N., Quinn M.A., Plebanski M. (2018). Immunotherapeutic Interleukin-6 or Interleukin-6 Receptor Blockade in Cancer: Challenges and Opportunities. Curr. Med. Chem..

[7] Marin-Acevedo J.A., Kimbrough E.O., Lou Y.Y. (2021). Next generation of immune checkpoint inhibitors and beyond. J. Hematol. Oncol..

[8] Scarfo I., Maus M.V. (2017). Current approaches to increase CAR T cell potency in solid tumors: Targeting the tumor microenvironment. J. Immunother. Cancer.

[9] Albers P., Albrecht W., Algaba F., Bokemeyer C., Cohn-Cedermark G., Fizazi K., Horwich A., Laguna M.P., Nicolai N., Oldenburg J. (2015). Guidelines on Testicular Cancer: 2015 Update. Eur. Urol..

[10] Oosterhuis J.W., Looijenga L.H. (2005). Testicular germ-cell tumours in a broader perspective. Nat. Rev. Cancer.

[11] Groll R.J., Warde P., Jewett M.A.S. (2007). A comprehensive systematic review of testicular germ cell tumor surveillance. Crit. Rev. Oncol. Hemat.

[12] Seidel C., Daugaard G., Nestler T., Tryakin A., Fedyanin M., Fankhauser C., Hermanns T., Aparicio J., Heinzelbecker J., Paffenholz P. (2020). Human chorionic gonadotropin-positive seminoma patients: A registry compiled by the global germ cell tumor collaborative group (G3). Eur. J. Cancer.

[13] Hvarness T., Nielsen J.E., Almstrup K., Skakkebaek N.E., Rajpert-De Meyts E., Claesson M.H. (2013). Phenotypic characterisation of immune cell infiltrates in testicular germ cell neoplasia. J. Reprod. Immunol..

[14] Klein B., Haggeney T., Fietz D., Indumathy S., Loveland K.L., Hedger M., Kliesch S., Weidner W., Bergmann M., Schuppe H.C. (2016). Specific immune cell and cytokine characteristics of human testicular germ cell neoplasia. Hum. Reprod..

[15] Siska P.J., Johnpulle R.A.N., Zhou A., Bordeaux J., Kim J.Y., Dabbas B., Dakappagari N., Rathmell J.C., Rathmell W.K., Morgans A.K. (2017). Deep exploration of the immune infiltrate and outcome prediction in testicular cancer by quantitative multiplexed immunohistochemistry and gene expression profiling. Oncoimmunology.

[16] Boutilier A.J., Elsawa S.F. (2021). Macrophage Polarization States in the Tumor Microenvironment. Int. J. Mol. Sci..

[17] Zhao X., Wei Y.Q., Kariya Y., Teshigawara K., Uchida A. (1995). Accumulation of gamma/delta T cells in human dysgerminoma and seminoma: Roles in autologous tumor killing and granuloma formation. Immunol. Investig..

[18] Balzer B.L., Ulbright T.M. (2006). Spontaneous regression of testicular germ cell tumors: An analysis of 42 cases. Am. J. Surg. Pathol..

[19] Nestler T., Dalvi P., Haidl F., Wittersheim M., Von Brandenstein M., Paffenholz P., Wagener-Ryczek S., Pfister D., Koitzsch U., Hellmich M. (2022). Transcriptome analysis reveals upregulation of immune response pathways at the invasive tumour front of metastatic seminoma germ cell tumours. Br. J. Cancer.

[20] Gayer F.A., Fichtner A., Legler T.J., Reichardt H.M. (2022). A Coculture Model Mimicking the Tumor Microenvironment Unveils Mutual Interactions between Immune Cell Subtypes and the Human Seminoma Cell Line TCam-2. Cells.

[21] Dieckmann K.P., Skakkebaek N.E. (1999). Carcinoma in situ of the testis: Review of biological and clinical features. Int. J. Cancer.

[22] Fichtner A., Richter A., Filmar S., Gaisa N.T., Schweyer S., Reis H., Nettersheim D., Oing C., Gayer F.A., Leha A. (2021). The detection of isochromosome i(12p) in malignant germ cell tumours and tumours with somatic malignant transformation by the use of quantitative real-time polymerase chain reaction. Histopathology.

[23] Rodriguez S., Jafer O., Goker H., Summersgill B.M., Zafarana G., Gillis A.J.M., van Gurp R.J.H.L.M., Oosterhuis J.W., Lu Y.J., Huddart R. (2003). Expression profile of genes from 12p in testicular germ cell tumors of adolescents and adults associated with i(12p) and amplification at 12p11.2-p12.1. Oncogene.

[24] Nettersheim D., Westernstroer B., Haas N., Leinhaas A., Brustle O., Schlatt S., Schorle H. (2012). Establishment of a versatile seminoma model indicates cellular plasticity of germ cell tumor cells. Gene Chromosome Cancer.

[25] Jostes S.V., Fellermeyer M., Arevalo L., Merges G.E., Kristiansen G., Nettersheim D., Schorle H. (2020). Unique and redundant roles of SOX2 and SOX17 in regulating the germ cell tumor fate. Int. J. Cancer.

[26] Bahrami A.R., Matin M.M., Andrews P.W. (2005). The CDK inhibitor p27 enhances neural differentiation in pluripotent NTERA2 human EC cells, but does not permit differentiation of 2102Ep nullipotent human EC cells. Mech. Dev..

[27] Eini R., Stoop H., Gillis A.J., Biermann K., Dorssers L.C., Looijenga L.H. (2014). Role of SOX2 in the etiology of embryonal carcinoma, based on analysis of the NCCIT and NT2 cell lines. PLoS ONE.

[28] Nettersheim D., Heimsoeth A., Jostes S., Schneider S., Fellermeyer M., Hofmann A., Schorle H. (2016). SOX2 is essential for in vivo reprogramming of seminoma-like TCam-2 cells to an embryonal carcinoma-like fate. Oncotarget.

[29] de Jong J., Stoop H., Gillis A.J.M., Hersmus R., van Gurp R.J.H.L.M., de Geijn G.J.M.V., van Drunen E., Beverloo H.B., Schneider D.T., Sherlock J.K. (2008). Further characterization of the first seminoma cell line TCam-2. Gene Chromosome Cancer.

[30] Eckert D., Nettersheim D., Heukamp L.C., Kitazawa S., Biermann K., Schorle H. (2008). TCam-2 but not JKT-1 cells resemble seminoma in cell culture. Cell Tissue Res..

[31] Bremmer F., Bohnenberger H., Kuffer S., Oellerich T., Serve H., Urlaub H., Strauss A., Maatoug Y., Behnes C.L., Oing C. (2019). Proteomic Comparison of Malignant Human Germ Cell Tumor Cell Lines. Dis. Markers.

[32] Batool A., Chen S.R., Liu Y.X. (2019). Distinct Metabolic Features of Seminoma and Embryonal Carcinoma Revealed by Combined Transcriptome and Metabolome Analyses. J. Proteome Res..

[33] Bremmer F., Hemmerlein B., Strauss A., Burfeind P., Thelen P., Radzun H.J., Behnes C.L. (2012). N-cadherin expression in malignant germ cell tumours of the testis. BMC Clin. Pathol..

[34] Lunney J.K. (1998). Cytokines orchestrating the immune response. Rev. Sci. Tech..

[35] Fisher D.T., Appenheimer M.M., Evans S.S. (2014). The two faces of IL-6 in the tumor microenvironment. Semin. Immunol..

[36] Klein B., Schuppe H.C., Bergmann M., Hedger M.P., Loveland B.E., Loveland K.L. (2017). An in vitro model demonstrates the potential of neoplastic human germ cells to influence the tumour microenvironment. Andrology.

[37] Russell S.M., Lechner M.G., Mokashi A., Megiel C., Jang J.K., Taylor C.R., Looijenga L.H., French C.A., Epstein A.L. (2013). Establishment and characterization of a new human extragonadal germ cell line, SEM-1, and its comparison with TCam-2 and JKT-1. Urology.

[38] de Jong J., Stoop H., Gillis A.J., van Gurp R.J., van Drunen E., Beverloo H.B., Lau Y.F., Schneider D.T., Sherlock J.K., Baeten J. (2007). JKT-1 is not a human seminoma cell line. Int. J. Androl..

[39] Desmousseaux T., Arama E., Maxwell F., Ferlicot S., Hani C., Fizazi K., Lebacle C., Loriot Y., Boumerzoug M., Cohen J. (2022). Ultrasound and Magnetic Resonance Imaging of Burned-Out Testicular Tumours: The Diagnostic Keys Based on 48 Cases. Cancers.

