# Supplementary material for: The Subtype Identity of Testicular Cancer Cells Determines Their Immunostimulatory Activity in a Coculture Model
Source: Cancers (Basel). 2023 May 5;15(9):2619. doi: 10.3390/cancers15092619 (PMC10177190; doi:10.3390/cancers15092619)
Supplement: Supplementary file 1 [file cancers-15-02619-s001.zip › cancers-2326704-supplementary.pdf]

Table S1. Primer sequences

Gene	Forward primer	Reverse primer
18SRNA	*TCC AGG TCT TCA CGG AGC TTG TT*	*GGA TGT AAA GGA TGG AAA ATA CA*
CD25	*GTG GTG GGG CAG ATG GTT TA*	*TTG TGA CGA GGC AGG AAG TC*
CD69	*ACG CAG GTA GAG AGG AAC AC*	*ACC CTG TAA CGT TGA ACC AGT*
CD163	*GCA GTT TCC TCA AGA GGA GAG AA*	*ATG GCC TCC TTT TCC ATT CCA*
CD206	*CGA TCC GAC CCT TCC TTG ACT*	*AGT ATG TCT CCG CTT CAT GCC*
CDK4	*ATG GCT ACC TCT CGA TAT GAG C*	*CAT TGG GGA CTC TCA CAC TCT*
GDF3	*TGC TGT AAA ACT AAC CAA GGC TC*	*ACA GTA GAT CCT CCA ACC CC*
IFNG	*CTG TAA CTG CCA GGA CCC AT*	*TCT GTC ACT CTC CTC TTT CCA*
IL1B	*AGC CAT GGC AGA AGT ACC TG*	*TGA AGC CCT TGC TGT AGT GG*
IL2	*TTT ACA TGC CCA AGA AGG CCA*	*GCA CTT CCT CCA GAG GTT TG*
IL6	*TCA ATA TTA GAG TCT CAA CCC CCA*	*TTC TCT TTC GTT CCC GGT GG*
KI67	*TCC TTT GGT GGG CAC CTA AGA CCT G*	*TGA TGG TTG AGG TCG TTC CTT GAT G*
NANOG	*GAT TTG TGG GCC TGA AGA AA*	*AAG TGG GTT GTT TGC CTT TG*
OCT3/4	*CGA AAG AGA AAG CGA ACC AG*	*GCC GGT TAC AGA ACC ACA CT*
PERF1	*GGG ATT CCA GAG CCC AAG TG*	*GTG TGT CCA CTG GGA AGG AG*
SALL4	*TCC AGT TTA GCA CAA AGG GCA*	*GGA GGA AAA TGA CGT CTG GC*
SOX2	*ATG CAC CGC TAC GAC GTG A*	*CTT TTG CAC CCC TCC CAT T*
SOX17	*GGC GCA GCA GAA TCC AGA*	*CCA CGA CTT GCC CAG CAT*
TNFA	*ATG GCC TCC CTC TCA TCA GT*	*CTT GGT GGT TTG CTA CGA CG*
